# DAND5 Inactivation Enhances Cardiac Differentiation in Mouse Embryonic Stem Cells

**DOI:** 10.3389/fcell.2021.629430

**Published:** 2021-04-13

**Authors:** José Manuel Inácio, João von Gilsa Lopes, Ana Mafalda Silva, Fernando Cristo, Sara Marques, Matthias E. Futschik, José António Belo

**Affiliations:** ^1^iNOVA4Health, CEDOC, NOVA Medical School, NMS, Universidade Nova de Lisboa, Lisbon, Portugal; ^2^Faculty of Medicine, School of Public Health, Imperial College London, Medical School, St. Mary’s Hospital, London, United Kingdom

**Keywords:** cardiomyocyte proliferation, cardiac progenitor cell, cardiac differentiation, *Dand5*, embryonic stem cells

## Abstract

Deciphering the clues of a regenerative mechanism for the mammalian adult heart would save millions of lives in the near future. Heart failure due to cardiomyocyte loss is still one of the significant health burdens worldwide. Here, we show the potential of a single molecule, DAND5, in mouse pluripotent stem cell-derived cardiomyocytes specification and proliferation. *Dand5* loss-of-function generated the double of cardiac beating foci compared to the wild-type cells. The early formation of cardiac progenitor cells and the increased proliferative capacity of *Dand5* KO mESC-derived cardiomyocytes contribute to the observed higher number of derived cardiac cells. Transcriptional profiling sequencing and quantitative RT-PCR assays showed an upregulation of early cardiac gene networks governing cardiomyocyte differentiation, cell cycling, and cardiac regenerative pathways but reduced levels of genes involved in cardiomyocyte maturation. These findings prompt DAND5 as a key driver for the generation and expansion of pluripotent stem cell-derived cardiomyocytes systems with further clinical application purposes.

## Introduction

Cardiomyocyte loss is a significant process underlying heart failure prevalence in our aging society (Virani et al., 2020). From inherited cardiomyopathies, myocardial infarction to oncological treatments, the causes for cardiomyocyte death are vast, and its regeneration scarce or null (Leone et al., 2015; Yuan and Braun, 2017). The natural replication of cardiomyocytes is present during mammalian embryo and fetus development, but stops irreversibly within the first weeks of postnatal life (Payan et al., 2020). Cardiac development starts on both sides of the embryo, with cells from the heart-forming regions migrating from the anterior region of the primitive streak to the midline, forming the heart tube (Buckingham et al., 2005; Kelly et al., 2014; Meilhac et al., 2015). After a series of coordinated cell movements, the heart tube loops and fuses to form the 4-chamber structured heart. With the gradual differentiation of cardiac cells, cycles of contraction and relaxation, related to heart function, emerge consistently, allowing the entire organism’s blood supply. Thus, for cardiac morphogenesis and function, the specification, proliferation, migration, differentiation, and maturation of the heart precursor cells are essential. These processes require a precise spatial and temporal coordination of several signaling pathways at multiple levels since many of them are involved in more than one molecular mechanism and sometimes with opposite regulatory readouts (Brand, 2003; Olson et al., 2006; Bruneau, 2013; Paige et al., 2015). Over the last decade, we have expanded our knowledge on the regulatory network that is essential for cardiac development, regeneration, and remodeling during disease, namely due to the advances in “omics” technologies (O’Meara et al., 2015; Uosaki et al., 2015; Liu et al., 2019; Wang et al., 2020; Xiong and He, 2020; Zhou and Wang, 2020). During mesoderm specification, the T-box transcription factors T and EOMES activate the expression of the early cardiac transcription factor MESP-1 while repressing genes related to pluripotency (Bondue and Blanpain, 2010; Devine et al., 2014). Subsequently, the GATA zinc-finger family (e.g., GATA4), the T-box protein family (TBX5), the NK homeodomain family (NKX2-5), the MADS-box family (MEF2, SRF), are triggered to promote the differentiation of cardiac progenitor cells (Lien et al., 1999; Bruneau et al., 2001; Gottlieb et al., 2002; Dodou et al., 2004; Phan et al., 2005; Qian et al., 2012). In addition, the isoform switching of contractile genes, such as the later expression cardiac troponin T3 in comparison with the fetal cTnT1 isoform, plays a role in the maturation of the cardiomyocytes (Taegtmeyer et al., 2010; Ames et al., 2013). Nevertheless, even with all the datasets and information currently available, identifying key regulatory genes and developmental gene networks remains a challenge.

DAND5 is an extracellular protein belonging to the family of TGF-β/Nodal signaling antagonists Cerberus/DAN (Belo et al., 2000, 2009). DAND5-mediated antagonism of Nodal signaling requires DAND5 binding to the ligand Nodal, and most likely, to Nodal (co)receptors, which consequently prevents agonist-receptor interaction and subsequent signaling activation. Furthermore, some Cerberus/Dan family members, including DAND5, are multivalent antagonists that also bind to and inhibit BMP and Wnt ligands (Belo et al., 2009). Interestingly, we have reported that loss-of-function of DAND5 in mice leads to a massive increase of the ventricular heart wall’s thickness caused by an increased mitotic index of the cardiomyocytes (CMs) at the compact myocardium (Araújo et al., 2014; Belo et al., 2017). Allied to these, increased levels of phosphorylated-SMAD2 and increased *Ccnd1* expression levels were detected in the hearts of *Dand5* knockout (*Dand5* KO) neonatal mice (Araújo et al., 2014). Although the significant mortality rate observed in *Dand5* knockout newborn mice is linked with the increased mitotic index of the cardiomyocytes, the notion that the proliferative and regenerative capacity of cardiac cells from the diseased heart can be stimulated by the modulation of a single endogenous signaling antagonist is exciting and open new therapeutic avenues.

To explore this hypothesis and further clarify the function of DAND5 in the molecular control of cardiomyogenesis, we successfully derived a *Dand5* KO mouse embryonic stem cell line that proved to be a valuable *in vitro* cardiac differentiation model. Using this cellular tool, we show that *Dand5* loss-of-function dramatically increases the proportion of FLK-1^+^/PDGFR-α^+^ cardiac progenitor cells. In addition, the knockout of *Dand5* activates cell-cycle regulators, augmenting cardiomyocyte proliferation. Furthermore, we provide regulatory information on the signaling pathways enriched in *Dand5* KO mESC-derived cardiomyocytes. In conclusion, the modulation of *Dand5* expression levels seems to play an important role in the output of cardiomyocytes derived from pluripotent stem cell systems. Thus, the modulation of DAND5 levels could be used for the generation of better mature-ready iPS-CM for use in cell therapies to heal a diseased heart.

## Materials and Methods

### Mice

The animals were maintained at 22 ± 1°C in a 12-h light-dark cycle. The mouse line used in this work was the *Dand5* knockout (*Dand5* KO) generated using E14 embryonic stem cells and currently in an 129 background (Marques et al., 2004). Embryonic stage E0.5 was considered at noon of the plugs detection day. All animal experiments were performed in accordance with the European Union (EU) guidelines for animal research and welfare, and in compliance with the Portuguese law and approved by the Consultative Commission of the Veterinary Agency from Portuguese Ministry of Agriculture (Directive 2010/63/EU of the European Parliament). All animal experiments were conducted under DGAV Permit No. 0421/000/000/2016.

### Derivation and Primary Culture of Mouse *Dand5* KO ESC Lines

Three days after vaginal plug detection, pregnant females were sacrificed by cervical dislocation and the uterine horns were surgically removed and immediately placed on a pre-heated M2 culture medium (EmbryoMax^®^, Millipore). The blastocysts were flushed, washed, and incubated with Acidic Tyrode’s solution [137 mM NaCl; 2.7 mM KCl; 1.6 mM CaCl_2_.2H_2_O; 0.5 mM MgCl_2_.6H_2_O; 5.6 mM glucose; 0.4% Polyvinylpyrrolidone (PVP), pH 2.5] to remove the zona pellucida.

One blastocyst was plated per 6-well plate well, on mitotically inactivated MEF feeders, containing ES cell medium composed by Knockout-DMEM medium (Thermo Fisher Scientific) supplemented with 15% FBS (HyClone, UT, United States), 1% MEM Non-Essential Amino Acids (Thermo Fisher Scientific), 1% Penicillin/Streptomycin, 2 mM L-glutamine, and 0.1 mM β-Mercaptoethanol (Thermo Fisher Scientific). In order to maintain pluripotency conditions, 1000 U mouse LIF (ESGRO^®^ Millipore) was added as well as 1 μM PD0325901 (Calbiochem^®^ Millipore) and 2 μM CHIRON99021 (Calbiochem^®^ Millipore) inhibitors. Blastocysts were incubated at 37°C, 5% CO_2_ for 48 h, avoiding any disturbance during this period to allow its attachment to the feeder layer. On day 3 after flushing, half of the medium was replenished. During days 3–7, the outgrowths were monitored daily, and the medium was renewed every day.

At day 7, the cells were dissociated and transferred onto new inactivated feeder layers. The incipient mESC line is at Passage 1 (P1), and the medium was daily renewed. After 3–4 days, mESC colonies started to be distinguishable.

### Karyotyping

*Dand5* KO mESC lines chromosome analysis was performed using GTG high-resolution banding technique by the Department of Genetics, Faculty of Medicine of the University of Porto – São João Hospital. In total, 15 metaphases were analyzed for each ES cell line.

### mESCs Differentiation Through Embryoid Bodies Formation

Undifferentiated *Dand5* KO and the respective E14 WT control mESC lines were used to test cells pluripotency potential and spontaneous differentiation by the hanging droplet method (Bover et al., 2018). Briefly, cells were dissociated into a single cell suspension and resuspended in fresh mESC medium without LIF. Then, mESCs were cultured in hanging drops (500 cells per droplet) for 48 h until the formation of embryoid bodies (EBs). Then, the EBs were cultured in static suspension until day 5, followed by adherent culture in 0.1% gelatin-coated wells up to day 10, prompting spontaneous differentiation. The culture medium was renewed every day.

### RNA Isolation for cDNA Synthesis and RT-qPCR

Total RNA was extracted from mouse mESCs, MEF cells, and EBs, at several days of differentiation, using TRI Reagent^®^ (Sigma) and the Direct-zol^TM^ RNA MiniPrep Kit (Zymo Research) according to the manufacturer’s instructions. The RNA samples were evaluated relating to quantity and quality using a spectrophotometer (Nanodrop 2000, Thermo Fisher Scientific). Only samples with 260/280 nm and 260/230 nm ratios equal or superior to 2.0 were considered. First strand cDNA was synthesized through reverse transcription reaction using RevertAid Reverse Transcriptase, Oligo (dT) primers, RiboLock RNase Inhibitor, and dNTP (Thermo Fisher Scientific). RT-qPCR reactions were performed in triplicate using a SensiFAST SYBR Lo-ROX mix (BIOLINE) (the primers listed in Supplementary Table 1) on a 7300 Real-Time PCR system (Applied Biosystems). Relative quantification of expression was performed using the ddCt method (Bustin, 2000) and normalized to GAPDH as a housekeeping gene and with E14 mESC line as reference.

### Fluorescent Immunocytochemistry

Undifferentiated or differentiated mESCs were fixed in 4% paraformaldehyde, incubated with primary antibodies (diluted in 1x PBS, 1% Bovine Serum Albumin, 0.05% sodium azide solution) overnight at 4°C, listed in Supplementary Table 2, followed by appropriated secondary antibody incubation, overnight at 4°C. Nuclei were stained with DAPI at room temperature and cell images were acquired with Zeiss Axio Imager Z2 microscope or Zeiss LSM710 confocal microscope (Carl Zeiss). Images were taken in sequential mode and posteriorly adjusted in ImageJ.

### Flow Cytometry

To detect and quantify cardiac progenitor cells, EBs were dissociated into a single cell suspension and incubated with the following antibodies: Phycoerythrin (PE)- conjugated Flk-1 (eBioscience; 1:50), Allophycocyanin (APC)-conjugated-PdgfR-α (eBioscience; 1:100) at 4°C for 30 min.

To analyze cardiomyocyte proliferation, newly synthesized DNA was labeled by incubating EBs with EdU for 90 min. EdU detection was done following the Click-iT EdU Alexa Fluor 488 Imaging Kit (Thermo Fisher Scientific) instructions and then incubated with antibodies for MLC2v at 4°C for 30 min. Relative fluorescence intensity of cells was detected by Becton Dickinson FACSCanto II (BD Biosciences). Analysis of results was performed using FlowJo v10 software (BD). A minimum of 30,000 events were acquired for each condition.

### RNA-Sequencing

Total RNA was extracted from three biological replicates within each time point as mentioned above. Libraries were constructed using Stranded mRNA Library Prep Kit. Pair-end libraries were sequenced on an Illumina PE150 Platform with an output of ∼40 M reads per sample. The quality of the reads was assessed using FastQC software and mapping of reads was performed applying the STAR (version 2.7.5c) aligner using murine genome from GENCODE Release M25 as reference (Dobin and Gingeras, 2015). In particular, the primary genome sequence assembly GRCm38 together with corresponding annotations was used. To obtain the read counts per gene, the featureCount function of the Bioconductor package Rsubread (version 2.2.6) was executed (Liao et al., 2019a). As a measure of gene expression, the transcripts per million (TPM) were subsequently calculated. Finally, the analysis of differential gene expression was performed using Bioconductor edgeR package (version 3.30.3) with a biological coefficient of variation of 0.2 (McCarthy et al., 2012). *P*-value correction for multiple testing was performed using the Benjamini Hochberg (FDR) method. Mappings of Ensembl IDs to gene symbols were extracted from Bioconductor package org.Mm.eg.db for murine genome annotation (version 3.11.4). As functional enrichment analysis for KEGG pathway categories, Over-Representation Analysis of differentially expressed genes was conducted using WebGestalt (Liao et al., 2019b). As input, differentially expressed genes with FDR lower than 0.01 and with either positive or negative logged (base 2) fold changes larger than 2 or smaller than −2 were selected. Using genes that associated with Heart Cardiomyopathy and Dilated Cardiomyopathy terms of KEGG, a heat map was produced based on Z Scores, which were calculated by subtracting the overall average log2 TPM from the log2 TPM of the respective sample, and dividing that result by the standard deviation of all log2 TPM values across all samples.

### Statistical Analysis

Statistical Analysis was performed using GraphPad Prism 7 software (GraphPad Software, Inc.; San Diego, CA, United States). All the experimental values are reported as mean ± SD.

In the case of the RT-PCR experiments, statistical differences between the two groups (mutant and control groups) were determined by applying the unpaired Student’s *t*-test. Also, a one-way ANOVA test was applied when more than two groups were compared. To reject the null hypothesis, the probability values of *p < 0.05. were considered statistically significant.

## Results

### Generation and Characterization of *Dand5* KO mESC Line

**FIGURE 1:**
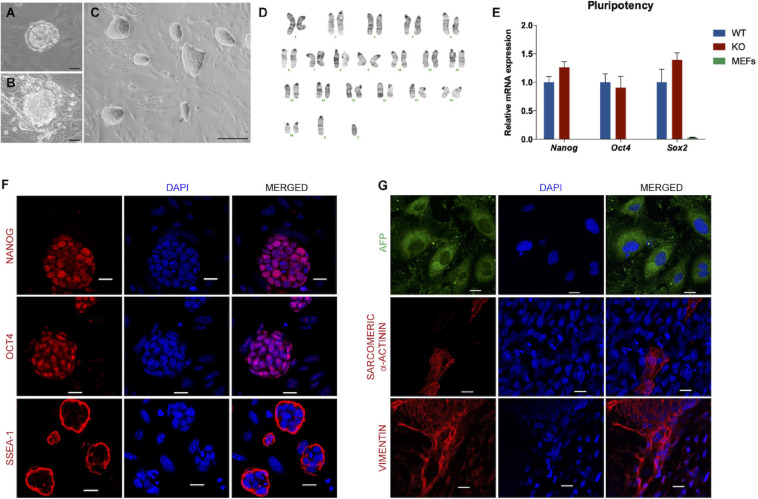
Characterization of the *Dand5* knockout mouse embryonic stem cell line. **(A)** Morphology of mouse blastocyst at stage E3.5, with well-defined ICM and blastocyst cavity; **(B)** Morphology of blastocyst hatched on MEF feeder at day 3 after collection; **(C)** Morphology of *Dand5* KO mESC line colonies cocultured with MEFs (scale bar: 100 μm); **(D)** Karyotype of a representative metaphase showing normal 40 chromosomes (XY); **(E)** Expression of *Nanog*, *Oct4*, and *Sox2* of the derived mouse *Dand5* KO ES cells by qRT-PCR (*n* = *3*). Experiments were performed in triplicate for each one. The expression level of each pluripotency gene is represented relative to the referred E14 ESC line, used as a positive control; **(F)** Immunofluorescence analysis show positive expression of NANOG, OCT4, and SSEA-1 in *Dand5* KO mESCs (scale bars: 20 μm); **(G)** The presence of AFP (endoderm), α-Actinin sarcomeric (mesoderm), and Vimentin (ectoderm) markers were positively detected at day 10 of differentiation by immunofluorescence analysis (scale bars: 20 μm).

To uncover the role of DAND5 in the mechanisms of cardiac mesoderm differentiation and in the number of *in vitro* produced cardiomyocytes, we successfully derived a mouse ES cell line from *Dand5* knockout (*Dand5* KO) mouse blastocysts. To access the blastocysts, pregnant females were sacrificed at stage E3.5, and the uterus was flushed, allowing the collection and handling of embryos (Figure 1A). The blastocysts were then incubated with Acidic Tyrode’s solution to disrupt the zona pellucida (Khalifa et al., 1992), which promotes blastocyst hatching. The mouse blastocysts were then plated in feeder cells and maintained in culture until clusters of outgrowths started to be visible (Figure 1B). Since the first passage, the *Dand5* KO mESCs colonies adopted an oval morphology with clear light boundaries, and within cells showed a large nucleus compared with a reduced cytoplasm (Figure 1C). *Dand5* KO mESCs were cocultured with freshly MEFs feeders up to passage 3, which promoted an efficient derivation and pluripotency maintenance. For subsequent passages, the cells were cultured on 0.1% gelatin-coated plates.

To ensure *Dand5* KO mESCs quality and purity, we performed a full characterization of the undifferentiated cells relating to its chromosomic and genetic stability, pluripotency, and differentiation capacities. Firstly, to authenticate the new *Dand5* KO mESCs, we successfully confirmed the knockout genotype of the derived cells according to what was established before (Marques et al., 2004; Supplementary Figure 1A). In addition, the male gender identification resulted from the positive amplification of a specific region of the *Sry* gene, classified as the master regulator involved in the early male phenotype (Polanco and Koopman, 2007), was also determined (Supplementary Figure 1B). Chromosomic integrity and stability were analyzed by karyotyping. In a total of 15 metaphases evaluated, we observed that *Dand5* KO mESC line presents a normal chromosomic number (40, XY), without any translocation detected. Likewise, the karyotype results confirmed the male gender, firstly identified by the PCR technique (Figure 1D).

To validate the pluripotency properties of the *Dand5* KO mESCs, we started by assessing the expression of pluripotency genes *Nanog*, *Oct4*, and *Sox2*, by quantitative RT-PCR at Passage 6. The results indicated that the *Dand5* KO mESC line shows expression levels comparable to the mESCs control cells for all these pluripotency genes (Figure 1E). In this experiment, RNA isolated from mouse embryonic fibroblast cells was used as a negative control, and as expected, these cells did not express any of these pluripotency genes. Then, protein expression for pluripotency markers was examined by immunofluorescence. The derived mESCs expressed nucleus markers – NANOG and OCT4 – and the surface marker SSEA-1 positively, confirming the stemness of the *Dand5* KO mESCs (Figure 1F). Testing the spontaneous differentiation capacity in the three germ layers is a crucial validation step to guarantee the pluripotency properties of any given ES cell line. To do so, we prepared cells to form embryoid bodies using the hanging drop differentiation. The results clearly show that some *Dand5* KO mESCs spontaneously differentiate to the endoderm lineage, positively marked with AFP, while others differentiate to the mesoderm lineage, positively marked with α-Actinin sarcomeric, and others differentiate to the ectoderm lineage, positively marked with Vimentin (Figure 1G). Altogether, these data demonstrated that we efficiently derivated and expanded an mESC line from blastocyst-stage *Dand5* KO embryos. The line shows pluripotent ground-state properties and capacity to differentiate into three germ layers from which all cell lineages that compose an organism derive.

### Cardiac Progenitor Numbers Are Increased During *Dand5* KO mESCs Cardiomyocyte Differentiation

**FIGURE 2:**
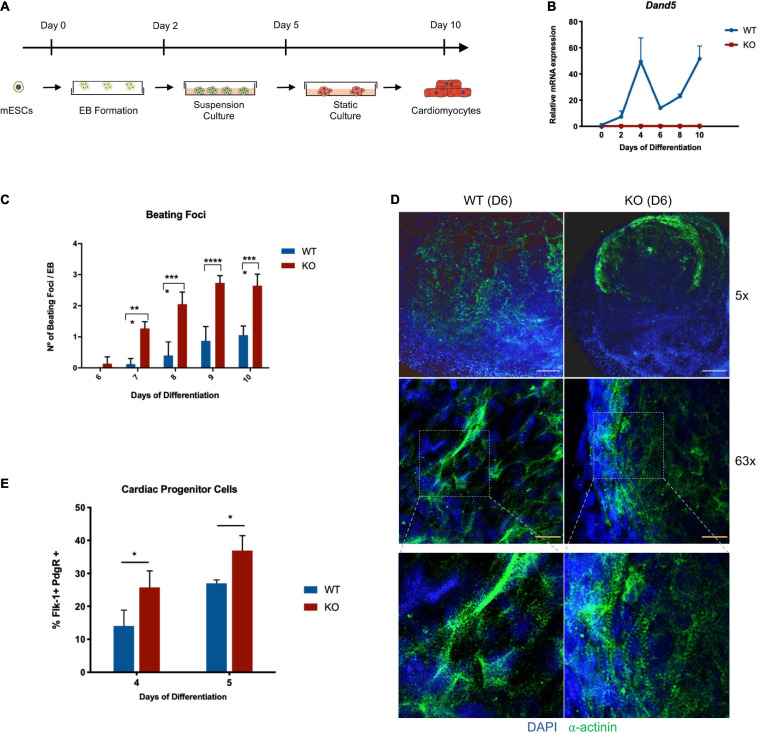
Analysis of *Dand5* KO mESCs during cardiomyocyte differentiation. **(A)** Schematic representation of the cardiomyocytes’ differentiation using the hanging drop technique; **(B)** Relative *Dand5* expression throughout cardiac differentiation. **(C)** Measurement of beating foci per KO and WT EBs. Results are expressed as the total number of beating foci with respect to the total number of plated EBs; **(D)** Whole-mount EB immunofluorescence analysis show positive expression of sarcomeric α-actinin on *Dand5* KO and WT EBs differentiated for 6 days. DAPI was used as a positive control for nuclear staining (scale bars: white 200 μm; yellow 20 μm). **(E)** Analysis of FLK-1 and PDGFR-α expression by flow cytometry. All the results represent the mean ± SD of three independent biological experiments. Unpaired Student’s *t*-test was applied to compare the differences between WT and KO groups in each day of differentiation. Statistically significant results were considered when *p < 0.05, ***p < 0.001, and ****p < 0.0001.

**FIGURE 3:**
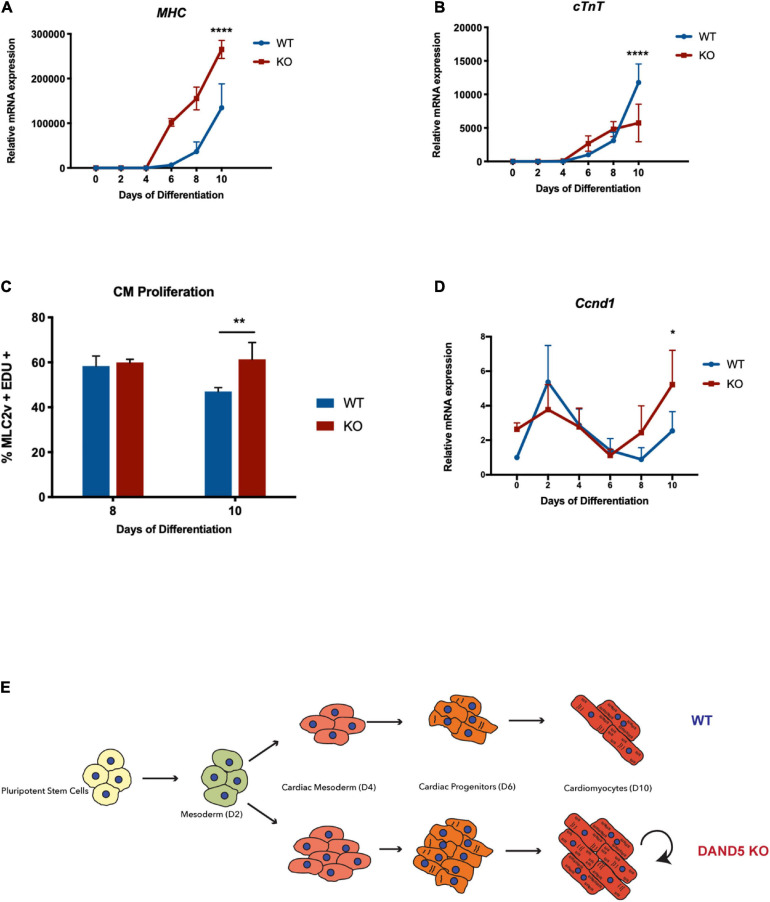
*Dand5* KO leads to increased cardiomyocyte proliferation **(A)** Relative mRNA expression of *MHC* in *Dand5* KO and WT differentiated cells; **(B)** Relative *cTNT* expression throughout cardiac differentiation *of Dand5* KO and WT cells; **(C)** Analysis of cardiomyocyte proliferation by labeling newly synthesized DNA using EdU incubation for 1.5 h. Relative fluorescence intensity of cells was analyzed by flow cytometry; **(D)** Relative mRNA expression of *Ccnd1* gene in *Dand5* KO and WT differentiated cells. **(E)** Summary of the results: the increased formation of cardiac progenitor cells and the increased proliferative capacity of *Dand5* KO mESC-derived cardiomyocytes contribute to the observed higher number of derived cardiac cells. All the results represent the mean ± SD of three independent biological experiments. Unpaired Student’s *t*-test was applied to compare the differences between WT and KO groups in each day of differentiation. Statistically significant results were considered when *p < 0.05, **p < 0.01, and ****p < 0.0001.

After the pluripotency properties and stability of the derived cell lines were confirmed, we used this model to study the role of DAND5 in cardiomyogenesis *in vitro*. The differentiation of pluripotent KO cells into cardiomyocytes was induced by the hanging drop method leading to Embryoid Bodies’ formation (EBs) (Figure 2A). This protocol was performed in three independent experiments using *Dand5* KO mESCs and E14 WT mESC lines control in parallel. Through a spontaneous differentiation process, cardiac cells originated with observable rhythmic beating foci along 10 days. Interestingly, beating foci areas started to be visible in the KO differentiated cells prior to the WT cell line (Figure 2C). At day 6, their contractile movements were notorious in all the plated EBs at different rhythmic rates, while in the control line, the beating foci areas start to show contractile movements only between days 7 and 8 in a sporadic number of EBs (Figure 2C). By performing the statistical analysis of the counted contractile areas, we observed that each KO EB originates the double of beating foci areas compared to the WT EBs during the entire differentiation protocol. From day 7 to day 10, the differences between the two cell lines were statistically significant, indicating that the mutant *Dand5* cells have a higher capacity to develop beating foci areas from the initial to the final stages of differentiation. To confirm that this observed phenotype results in earlier differentiation and organization of cardiomyocytes, we performed an immunostaining against sarcomeric α-actinin (Figure 2D). At day 6, Dand5 KO mESCs showed clear and organized α-actinin positive foci while the control line displayed a scattered signal. Additionally, defined sarcomeric structures’ appearance indicated that the cardiac program is enhanced in Dand5 KO cells (Figure 2D). This earlier and higher activation of cardiomyocyte differentiation was confirmed by α*-MHC* expression, already upregulated at day 6 of differentiation (Figure 3A). These results suggest that DAND5 influences the commitment, number, and electrophysiological capabilities of cardiac progenitor cells (CPCs). To determine whether the loss-of-function of the *Dand5* gene could promote an early formation of CPCs, we analyzed the population of FLK-1^+^/PDGFR-α^+^ cells from both *Dand5* KO and WT EBs by FACS. At day 4, we found that *Dand5* KO EBs have ∼40% more cardiovascular precursors cells than the WT EBs (Figure 2E). While there was an expected increase in the numbers of FLK-1^+^/PDGFR-α^+^ cells on both DAND5 KO and WT at day 5, we still observed significantly more FLK-1^+^/PDGFR-α^+^ cells in the Dand5 KO EBs when compared to the WT counterparts (Figure 2E).

**FIGURE 4:**
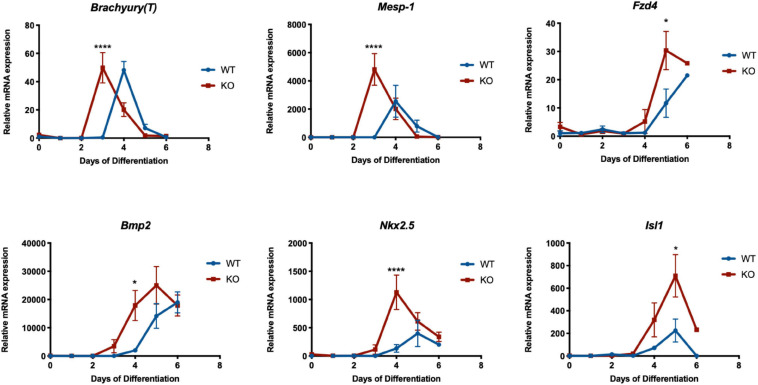
Relative mRNA expression of *Brachyury(T)*, *Mesp-1, Fzd4*, *Bmp2*, *Nkx2.5*, and *Isl1* genes in *Dand5* KO and WT differentiated cells. All the results represent the mean ± SD of three independent biological experiments. Unpaired Student’s *t*-test was applied to compare the differences between WT and KO groups in each day of differentiation. Statistically significant results were considered when *p < 0.05 and ****p < 0.0001.

Since the *Dand5* KO cell line differentiates into more CPC than the WT line, we investigated if the expression levels of cardiac mesoderm genes in the KO cell line are increased during the early stages of cardiomyocyte differentiation by quantitative RT-PCR. Firstly, we confirmed that *Dand5* is not expressed in the KO mESCs (Figure 2B). Cardiac-specific genes, including *Mesp-1*, *Isl1*, *Nkx2.5*, α*-MHC*, and *cTnT* along with *Brachyury(T)*, *Bmp2*, and *Fzd4* were assayed as markers to study the cardiac differentiation and specification along time. Interestingly, we observed an earlier expression pattern of almost all analyzed genes during the differentiation of the *Dand5* KO mESCs when compared to the WT mESC line (Figures 3, 4). High *Mesp-1* mRNA relative expression was found at day 3 of differentiation in the KO cells, and only at day 4 in the WT line. In addition, we observed that *Dand5* KO cells show a significantly higher peak of *Mesp-1* expression relative to the WT cells (Figure 4). This data suggests that in the absence of *Dand5*, ES cells demonstrate a faster and increased capacity for mesoderm formation toward the *Mesp-1* cardiogenic mesoderm lineage. Accordingly, *Brachyury(T)*, which is co-expressed along with *Mesp-1* in the mesodermal cells of the primitive streak (David et al., 2011), was also highly expressed in *Dand5* KO cells (Figure 4). The expression of *Fzd4*, a lateral plate mesoderm marker, was also higher in the KO cells when compared to the control line (Figure 4). The First Heart Field (FHF) progenitor’s marker, *Nkx2.5*, was significantly upregulated in the differentiated *Dand5* KO mESCs (Figure 4), marking the stage of cells’ commitment into the first myocardial lineage. Besides, levels of *Isl1* expression increased substantially at day 4, reaching the peak at day 5 for the mutant and the control lines (Figure 4). Expression of *Isl1* is commonly used to identify cells of the second Heart as the late cardiac progenitors to commit into the myocardial cells (Moretti et al., 2006). Comparing the relative levels of *Isl1* expression at day 5, the differentiated *Dand5* KO mESCs present higher gene expression levels than the control line (Figure 4). Taken together, these results corroborate the hypothesis that loss-of-function of the *Dand5* gene, in differentiating mESCs, increases the number of cardiac progenitors, which in turn results in a higher capacity to develop beating foci and, therefore, production of functional cardiomyocytes. Interestingly, *Dand5* displays two peaks of expression, at day 4 and by day 8–10 of differentiation (Figure 2B). Consequently, we can hypothesize that DAND5 may be relevant to regulate pathways involved in the specification and in the proliferation of cardiomyocytes at those two time-windows.

### *Dand5* KO Increases the Proliferation of ESC-Derived Cardiomyocytes

To examine the functionality and maturation state of the *Dand5* KO mESC-derived cardiomyocytes, we analyzed the expression of α-myosin heavy chain (α*-MHC)* and cardiac troponin T (*cTnT)* genes (Figure 3). Myosin is a protein that contributes to the generation of the contractile movements during the early mouse heart development through the mediation of ATP molecules (Ng et al., 2010). The qRT-PCR results identified an up-regulation of α*-MHC* gene expression in the KO line when compared to the WT line, already evident by day 6 and still persisting at day 10 of differentiation (Figure 3A). This result suggests again an increased stimulation cardiomyogenesis resulting from the loss-of-function of *Dand5*. In contrast, *cTnT* expression levels were significantly lower in the differentiating *Dand5* KO cells compared to WT cells at day 10 (Figure 3B). cTnT is one of the main regulatory proteins capable of controlling the ionic Ca^2+^ variations and anchor the other troponin components, essential for myocardium contraction (Nishii et al., 2008). Thus, this result indicated a possible structural commitment delay of the *Dand5* KO mESC-derived cardiomyocytes characterized by a reduced number of cTnT proteins at the sarcomere level and, consequently, impairment in their phenotypic maturation. Curiously, comparing *Bmp2* expression at CPC stage (day 4–5), we observed that *Dand5* KO cells have higher *Bmp2* expression than the WT (Figure 3E). It has been demonstrated that ectopic expression of *Bmp2* stimulates proliferation and blocks fully cardiomyocyte differentiation in embryoid bodies (Prados et al., 2018). To confirm the proliferation state of the *Dand5* KO mESC-derived cardiomyocytes, a proliferation assay consisting of labeling newly synthesized DNA with EdU and posterior fluorescence labeling using the Click-iT EdU Kit was performed at day 8 and day 10 of differentiation (Figure 3C). The results of this assay indicate that the knockout of DAND5 increases the proliferation of cardiomyocytes derived from the *Dand5* KO mESC line. Moreover, a statistically significant increase in the expression of the cardiac cell cycle regulator *Ccnd1* was found in the KO cells at day 10 (Figure 3D). This explains the observed increased levels of α*-MHC* and the decreased levels of *cTnT* expression in the differentiated *Dand5* KO cardiomyocytes. *cTnT* is usually upregulated in a more mature cardiomyocyte phenotype state. Therefore, our results suggest that *Dand5* KO mESC-derived cardiomyocytes sustain their self-renewal and immature state instead of long-term maturation, being capable to proliferate more than the WT line.

### Transcriptional Changes Toward a Cardiomyocyte Fate Are More Advanced in *Dand5* KO Cells

**FIGURE 5:**
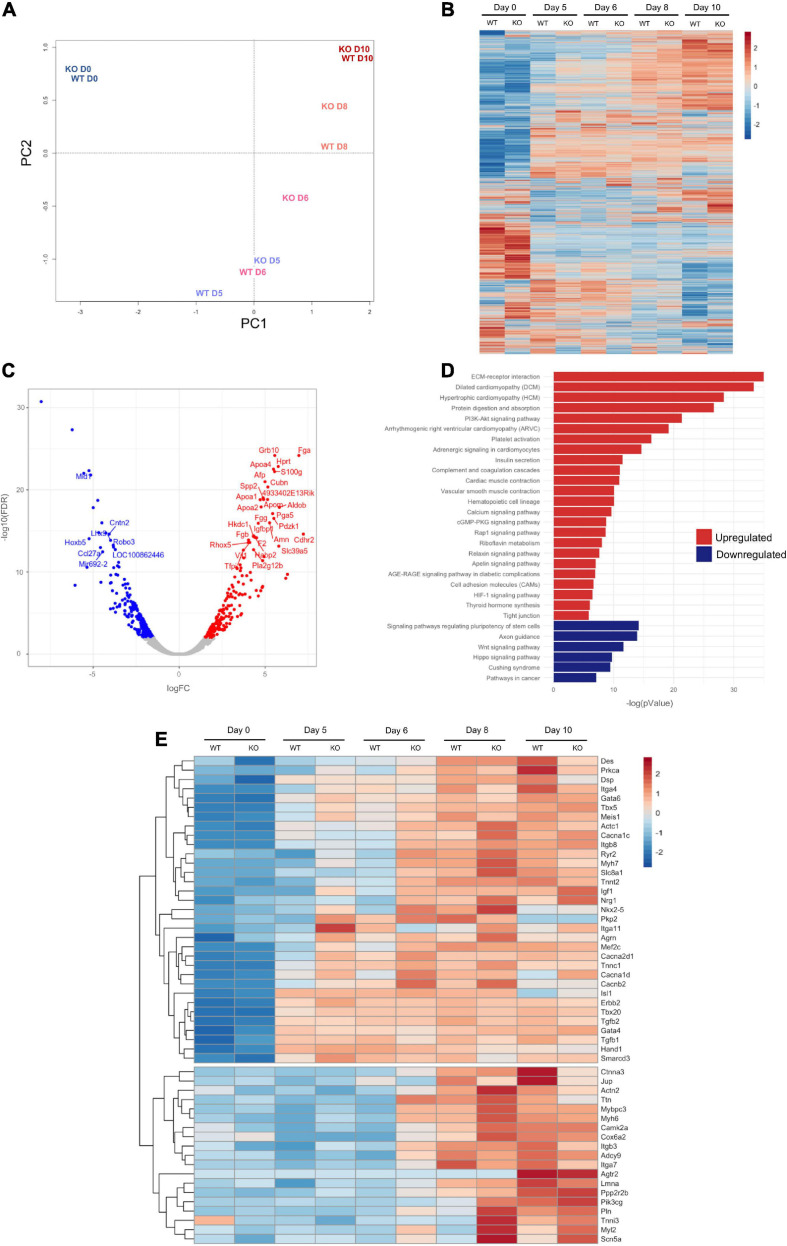
RNA-seq Analysis of EB differentiation up to day 10. **(A)** Principal Component Analysis (PCA) of WT and *Dand5* KO samples from EB differentiation from mESC (D0) to day 10 of differentiation (D10); **(B)** Heat Map of all the genes commonly counted in all the samples. Profiles of expression of each gene were hierarchically clustered; **(C)** Volcano plot of genes differentially expressed at day 6 of EB differentiation. Genes with FDR < 0.01 were colored in red. Results are relative to WT data. **(D)** Pathway enrichment analysis of genes differentially expressed at day 6 of EB differentiation. Results are relative to WT data; **(E)** Heat Map of genes related with Heart Cardiomyopathy and Dilated Cardiomyopathy terms of KEGG. Profiles of expression of each gene were hierarchically clustered.

To examine the difference between the global transcriptional profile of *Dand5* KO and WT mESC, we performed next generation RNA-sequencing (RNA-seq), and compared the transcriptomes of undifferentiated cells and differentiated cells at day 5 (EBs in suspension), day 6 (24 h after EB plating), day 8, and day 10. Principal component analysis (PCA) indicated that *Dand5* KO samples, except for the undifferentiated and day 10 cells, were consistently closer to WT samples of later days than the corresponding WT samples themselves (Figure 5A). This suggests that *Dand5* KO EBs progressed faster through cardiac mesoderm induction and differentiation. Next, we decided to confirm the existence of different expression patterns under the absence of DAND5 during EB differentiation on a gene level. We obtained an overall picture of the genetic expression across the time series by visualization and clustering of genes that were detected by RNA seq. As the resulting heat map displays, expression patterns of *Dand5* KO samples are distinct from their WT counterparts, mainly at days 5, 6, and 8 (Figure 5B). Differences can still be observed at day 10 samples, but not in the same degree as in the other days, which is in accordance with the PCA analysis (Figure 5A).

Focusing our analysis at day 6, when visible beating foci areas in the *Dand5* KO cells start to emerge, we generated a Volcano Plot and selected the genes with FDR lower than 0.01 and absolute log FC higher than 2 between *Dand5* KO and WT samples (Figure 5C). Then, a KEGG pathway enrichment analysis of the differentially expressed genes was carried out and suggested that upregulated differentially expressed genes were involved in pathways such as dilated cardiomyopathy, hypertrophic cardiomyopathy, PI3K-Akt signaling pathway, cardiac muscle contraction or calcium signaling pathway (Figure 5D). On the other hand, we observed that the downregulated differentially expressed genes were associated with pathways such as pluripotency maintenance, Wnt, or Hippo signaling (Figure 5D). This is in line with the expected results for *Dand5* KO differentiating cells, which were already committing to a cardiac lineage, with an increased proliferative capacity, while their WT counterparts are still delayed in this process.

Next, we evaluated a panel of well-studied genes involved in different cardiomyogenic processes in more detail. Notably, the clustered heat map for the differential expression (Figure 5E) reveals two distinct gene expression signatures that can be interpreted as two different transcriptional waves. The first cluster contains genes known to be related to early cardiac induction and being expressed before day 5, which explains the similar levels of expression for these genes (e.g., *Isl1* and *Hand1*). Genes related to early cardiomyocyte differentiation, cardiovascular morphogenesis, and function, as *Nkx2.5*, *Tbx5*, *Mef2c*, or *Tnnc1*, are also in the first cluster. These genes started being upregulated in *Dand5* KO compared to corresponding WT samples as early as day 5, and their relative upregulation persists until genes show full induction in the WT samples. These results are in agreement with the PCR measurements, validating the RNA-seq results. The transcriptome analyses also indicated that the knockout of *Dand5* changed the expression of genes associated with cardiovascular morphogenesis and function. Genes like *MHC* (*Myh7*), *cTnT* (*Tnnt2*), *TNNI* (*Tnni3*), *Myl2*, were upregulated in the absence of *Dand5*. The second cluster of genes displays a different expression pattern, with main differences in transcription arising at days 6 and 8. Genes in this cluster are related with cardiomyocyte commitment, such as *Titin* (*Ttn*), usually a marker of cardiomyocyte maturation, *Ryr2*, that encodes a Ca^2+^ channel, or *Itgb7*, and integrin subunit related with cardiomyocyte interaction with ECM and other cells. Additionally, we observed an up-regulation of genes related with cardiomyocyte proliferation, *Agrn1* at day 8, and of *Igf1* and *Nrg1* at day 10 on *Dand5* KO samples. These findings suggested an increased cell proliferation in the *Dand5* KO cells, which is in line with the results from the cardiomyocyte proliferation assay.

## Discussion

In this study, we found that suppression of DAND5 leads to an increase in the number of cardiac progenitor cells and augments the proliferative state of the mESCs-derived cardiomyocytes. During the differentiation of the *Dand5* KO mESCs, a significant difference in the number of beating foci areas in the mutant cells compared to the control line was observed. This result is concomitant with the detected upregulation of *Mesp-1*, *Isl1*, and *Nkx2.5* within the period of cardiac mesoderm formation. During *in vivo* cardiogenesis, MESP1 proteins start to be recruited to the mouse primitive streak at E6.5 (Meilhac et al., 2014). Its expression is transient during the phase of mesoderm formation, and there are about 150–250 MESP1^+^ progenitor cells that will contribute to cardiac morphogenesis (Meilhac et al., 2014; Chabab et al., 2016; Meilhac and Buckingham, 2018). In the case of DAND5 absence, our data predict that this number of cells is increased, leading to abnormal migration of cells to the heart and posterior high numbers of cardiac cells. In agreement, the high levels of relative *Nkx2.5* and *Isl1* expression in the *Dand5* mutant also revealed that the production of cardiac progenitors, derived from the First and Second Heart Fields, could be increased. The First Heart Field cardiac lineage, which can be identified through the positive expression of the *Nkx2.5* gene, contributes primarily to the formation of the linear heart tube and ultimately to the origin of the mature cardiac cells that will populate the left ventricle (Buckingham et al., 2005). Second Heart Field cells are often identified by the positive expression of *Isl1* being these cells the late cardiac progenitors for myocardial, endothelial, and smooth muscle cells (Moretti et al., 2006). Our results also demonstrated that the absence of DAND5 changes strikingly the population of FLK-1^+^/PDGFR-α^+^ cardiac progenitor cells in differentiating mESCs. Moreover, it is noted that the size of the mature heart will depend on how the number of cardiac progenitor cells scales up (Chabab et al., 2016). Combining these previous findings with our new results suggests that the increase of the *Mesp-1*, *Isl1*, and *Nkx2.5* expression during mouse cardiogenesis may coincide with a larger number of FHF and SHF progenitors capable of forming a robust myocardium tissue. These combined features could provide an explanation for the progressive increase of the trabecular and compact myocardium observed in the DAND5 null-mutant embryos (Araújo et al., 2014).

Cardiomyocytes are derived from the lateral plate mesoderm, which is composed of cells that migrated from the primitive streak toward both these anterior lateral regions of the embryo. This population of cells is characterized by *Fzd4* expression. The abundance of FZD4 is positively correlated with higher cardiomyocytes yield (Yoon et al., 2018). In our experiment, *Fzd4* expression was upregulated during the differentiation of *Dand5* KO mESCs, which is in line with the expanded cardiogenic capacity demonstrated by DAND5 mutant cells. The observed expression pattern of *Bmp2* was also interesting. In mice, ectopic expression of *Bmp2* in the chamber myocardium maintains the cardiomyocyte in a primitive and proliferative state leading to heart dysmorphogenesis and embryonic death (Prados et al., 2018). In contrast, the upregulation of *Bmp2* in the DAND5 mutant cells could contribute to the proliferation and expansion of the cardiac progenitor cells while not affecting cardiac differentiation. Indeed, levels of expression of genes essential for proper cardiomyocytes contraction (α*-MHC* and *cTnT*) demonstrated so. The high levels of relative α*-MHC* expression detected in the knockout cells are compatible with a high number of derived-cardiomyocytes. Moreover, these cells display enhanced sarcomere structures at early differentiation stages, explaining the previous contractility of the *Dand5* KO EBs. Curiously, the decreased levels of *cTnT* expression found in the differentiated *Dand5* KO mESCs at latter differentiation stages, indicates that the produced cardiomyocytes were not fully mature. This agrees with prior results that demonstrated a decrease in *cTnT* expression in the mutant mouse embryos at E13 and E15 (Araújo et al., 2014).

Cyclins and cyclin-dependent kinase (CDKs) are defined as regulatory molecules during embryonic cardiomyocyte division (Ikenishi et al., 2012). During this development process, it was reported that the Wnt/β-catenin pathway is capable of interfering in the proliferation capacity, mainly in the ventricular cardiomyocytes (Buikema et al., 2013). The recruitment of CCND1 and CCND2 (Cyclin D1 and Cyclin D2) proteins is one of the key triggers of the cardiac cell cycle. In the present data, we found a statistical increase of *Ccnd1* in the *Dand5* KO cells at day 10, explaining the high number of derived cardiomyocytes. Moreover, our proliferation assay also highlighted the difference between the *Dand5* KO mESC-derived cardiomyocytes’ proliferative capacity and the control cell line.

Transcriptomic analysis supported the notion that *Dand5* has a broad and important biological significance during cardiac development. Clear differences in the differentiation process were observed in PCA following knockdown of *Dand5*, especially a temporal shift toward an anticipated cardiac transcriptional program. Genes affected included those commonly related with cardiac morphogenesis and function underlying proliferation and differentiation of specific populations of heart precursor cells (Buckingham et al., 2005; Srivastava, 2006; Epstein, 2010; Ikenishi et al., 2012), such as cardiac transcription factors (e.g., *Nkx2.5*, *Isl1*), structural genes (*MHC*, *cTnT*), or cell cycle regulators (e.g., *Ccnd1*), which have been also validated by the RT-PCR results. Nevertheless, taking advantage of the power of genome-level approach, *Dand5* KO cells revealed significant changes in genes related to ECM-receptor interaction, dilated cardiomyopathy, hypertrophic cardiomyopathy, adrenergic signaling in cardiomyocytes, cardiac muscle contraction, calcium signaling, cell adhesion molecules, tight junctions, among other biological processes. Similarly, signaling pathways associated with cell-cycle regulation, the Hippo, IGF-PI3K-Akt, and Neuregulin were also altered. From all the factors involved in stimulating cardiomyocyte proliferation, *Meis1*, *Nrg*, and *Igf1*, were the ones that attained our attention. *Meis1* has been associated with *Hox* genes and found to be involved in the cell cycle regulation of cardiomyocytes (Paul et al., 2020). Cardiac-specific deletion of *Meis1* expression increases the proliferative capacity of the cardiomyocytes of newborn mice, whereas overexpression of *Meis1* decreases neonatal cardiomyocyte regenerative window (Mahmoud et al., 2013). *Meis1* was downregulated in *Dand5* KO mESCs, which prompted the deactivation of the regulatory mechanism of cardiomyocyte cell-cycle arrest and may have resulted in a continued division and proliferation of CMs. In contrast, *Nrg1* and *Igf1* were upregulated in *Dand5* KO embryoid bodies. Several studies have shown that the overexpression of *Nrg1* not only promotes cardiomyocyte proliferation in mice but also improves cardiac function after heart injury (D’Uva et al., 2015; Gemberling et al., 2015; Santoro and Sahara, 2015). Ectopic *Igf* signaling also fosters cardiomyocyte proliferation by increasing cell-cycle activity in adult mice cardiomyocytes (Samarel, 2002). *Igf1* expression was also increased in *Dand5* KO cells when compared to the control line. All these observations indicated that the obtained results of the transcriptional analysis are a valuable starting tool to explore the function of *Dand5* as a regulator during the cardiomyocyte-specific differentiation and proliferation.

In conclusion, we successfully derived and characterized a stable *Dand5* knockout mouse embryonic stem cell line with the purpose to uncover the first insights related to the role of DAND5 as an important endogenous regulator of the mechanisms controlling differentiation and proliferation of cardiomyocytes during the first stages of life. Moreover, our findings suggest that DAND5 drives distinctive transcriptional programs associated with the differentiation and proliferative networks of CMs that could be explored as a novel therapeutical approach for a diseased heart.

## Data Availability Statement

The datasets presented in this study can be found in online repositories. The names of the repository/repositories and accession number(s) can be found below: https://www.ebi.ac.uk/arrayexpress/, E-MTAB-9986.

## Ethics Statement

The animal study was reviewed and approved by the Veterinary Agency from Portuguese Ministry of Agriculture (DGAV), Portugal.

## Author Contributions

JI and JB conceived and designed the study. JI, JG, AS, FC, and SM performed the experiments. JI, JG, and MF analyzed the RNA-Seq data. JI, JG, FC, and JB wrote the original draft. All authors critically read and approved the final manuscript.

## Conflict of Interest

The authors declare that the research was conducted in the absence of any commercial or financial relationships that could be construed as a potential conflict of interest.
