# Supplementary material for: DAND5 Inactivation Enhances Cardiac Differentiation in Mouse Embryonic Stem Cells
Source: Front Cell Dev Biol. 2021 Apr 13;9:629430. doi: 10.3389/fcell.2021.629430 (PMC8078107; doi:10.3389/fcell.2021.629430)
Supplement: Supplementary file 1 [file Data_Sheet_1.docx]

Supplementary Material

**1A**


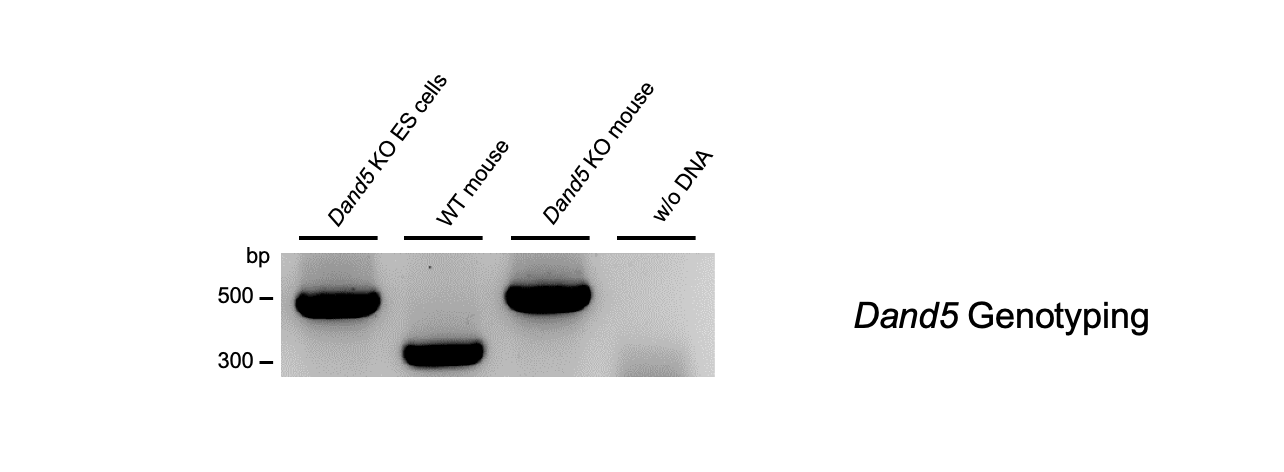


**Supplementary Figure 1A.** Mutant genotyping of the derived *Dand5* mES cells confirmed by PCR. Product bands with 500 bp size were detected in the derived *Dand5* mES cells and, also, in the adult KO mouse used as positive control. In opposition, the PCR product for the sample of the WT mouse resulted in a 300 bp band.

**1B**


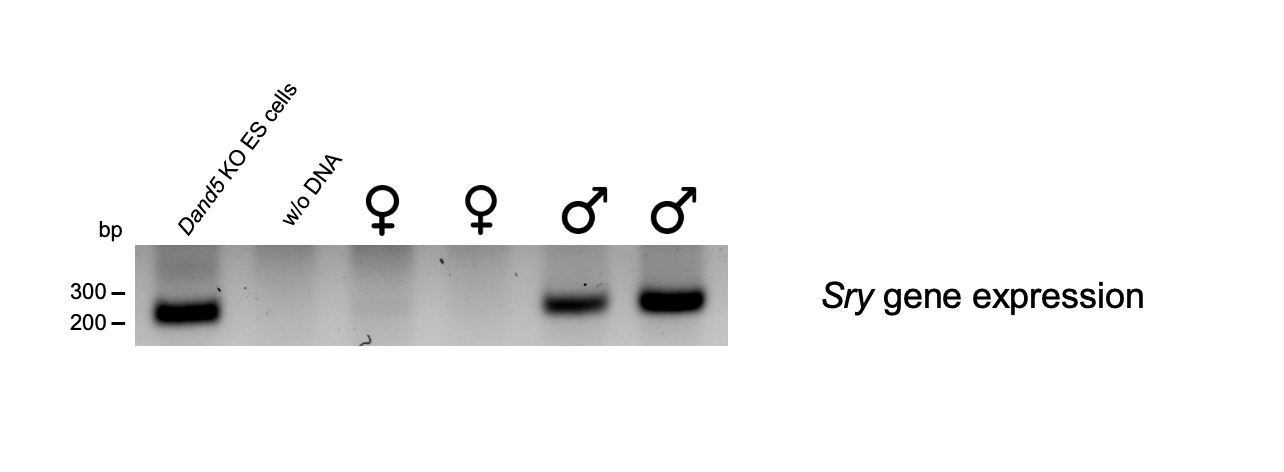


**Supplementary Figure 1B.** *Sry* gene expression revealed by PCR. A product band with 247 bp size was detected in male *Dand5* mES cells, also, in both male mouse controls.

**Table 1 – Primer List**

| **Gene** | **Sequence** | **Annealing Temperature (ºC)** |
| --- | --- | --- |
| *Nanog* | Fwd 5’ AGG GTC TGC TAC TGA GAT GCT CTG 3’  Rv 5’ CAA CCA CTG GTT TTT CTG CCA CCG 3’ | 61 |
| *Oct4* | Fwd 5’ AGT ATG AGG CTA CAG GGA CA 3’  Rv 5’ CAA AGC TCC AGG TTC TCT TG 3’ | 61 |
| *Sox2* | Fwd 5’ CGA GAT AAA CAT GGC AAT CAA ATG 3’  Rv 5’ AAC GTT TGC CTT AAA CAA GAC CAC 3’ | 56 |
| *Brachyury(T)* | Fwd 5’ AAC AGC TCT CCA ACC TAT GC 3’  Rv 5’ TAC CAT TGC TCA CAG ACC AG 3’ | 60 |
| *Mesp-1* | Fwd 5’ TGT ACG CAG AAA CAG CAT CC 3’  Rv 5’ TTG TCC CCT CCA CTC TTC AG 3’ | 61 |
| *Isl1* | Fwd 5’ CCT GTG TGT TGG TTG CGG CA 3’  Rv 5’ GGG CAC GCA TCA CGA AGT CG 3’ | 61 |
| *Dand5* | Fwd 5’ GCA GAG AGT AGC TGC TGG TGT GCC TTT3’  Rv 5’ CGG CAC ACA GCT GTT GCA GAA GAC TAC 3’ | 66 |
| *Ccnd1* | Fwd 5’ CAA ATG GAA CTG CTT CTG GTG AAC AA 3’  Rv 5’ GGA GGG TGG GTT GGA AAT GAA CTT 3’ | 63 |
| *cTnT* | Fwd 5’ GGA AAT CCA AGA TCA CTG CCT CC 3’  Rv 5’ GGG CAC TGA GGG ACA GAC CA 3’ | 60 |
| *Fzd4* | Fwd 5’ TGC CAG AAC CTC GGC TAC A 3’  Rv 5’ ATG AGC GGC GTG AAA GTT GT 3’ | 60 |
| *Nkx2.5* | Fwd 5’ CCA CTC TCT GCT ACC CAC CT 3’  Rv 5’ CCA GGT TCA GGA TGT CTT TGA 3’ | 60 |
| *α-Mhc* | Fwd 5’ GAT GGC ACA GAA GAT GCT GA 3’  Rv 5’ CTG CCC CTT GGT GAC ATA CT 3’ | 60 |
| *Bmp2* | Fwd 5’ TGA GGA TTA GCA GGT CTT TG 3’  Rv 5’ CAC AAC CAT GTC CTG ATA A 3’ | 61 |
| *Gapdh* | Fwd 5’ AAG AAG GTG GTG AAG CAG GC 3’  Rv 5’ GCC TCT CTT GCT CAG TGT CC 3’ | 61 |

**Table 2 – Antibody List**

| **Antibody** | **Dilution** | **Source** |
| --- | --- | --- |
| Nanog (rabbit) | 1:150 | Abcam |
| Oct4 (rabbit) | 1:150 | Abcam |
| SSEA-1 (mouse) | 1:50 | Abcam |
| AFP (goat) | 1:100 | Santa Cruz Biotechnology, Inc. |
| α-Actinin (Sarcomeric) (mouse) | 1:100 | Sigma |
| Vimentin (rabbit) | 1:50 | Sigma |
| MLC2v (rabbit) | 1:100 | ProteinTech |
| Alexa Fluor® 594 Donkey anti-mouse IgG | 1:300 | Jackson ImmunoResearch Laboratories |
| Alexa Fluor® 594 Donkey anti-rabbit IgG | 1:300 | Jackson ImmunoResearch Laboratories |
| Alexa Fluor® 488 Donkey anti-goat IgG | 1:300 | Jackson ImmunoResearch Laboratories |
| Alexa Fluor® 647 Donkey anti-rabbit IgG | 1:5000 | Jackson ImmunoResearch Laboratories |
